# Validation of the Spanish version of the MD Anderson symptom inventory – heart failure (MDASI-HF-Spanish) module

**DOI:** 10.1186/s40959-019-0055-4

**Published:** 2019-12-04

**Authors:** Anecita Fadol, Joaquin Buitrago, Maria C. Diaz, Valerie Shelton, Carolyn Harty, Tito R. Mendoza

**Affiliations:** 1 Department of Nursing, The University of Texas MD Anderson Cancer Center, 1400 Holcombe Boulevard, Unit 0456, Houston, TX 77030-4009 USA; 2 Nursing Education, The University of Texas MD Anderson Cancer Center, 1400 Holcombe Boulevard, Unit 0456, Houston, TX 77030-4009 USA; 3 Department of Symptom Research, The University of Texas MD Anderson Cancer Center, 1400 Holcombe Boulevard, Unit 0456, Houston, TX 77030-4009 USA

**Keywords:** Cancer, Heart failure, Linguistic validation, Spanish version, Symptom instrument

## Abstract

**Background:**

The lack of a validated symptom assessment instrument in Spanish for patients with cancer and heart failure (HF) can affect the care and impede the recruitment and participation of Spanish-speaking patients in clinical trials. Spanish is the second most common language spoken by the largest and most rapidly growing racial/ethnic minority group in the United States. To bridge the language barrier and improve symptom management in Spanish-speaking patients with cancer and HF, the MD Anderson Symptom Inventory-Heart Failure (MDASI-HF) was translated to Spanish (MDASI-HF- Spanish).

**Aim:**

To validate the MDASI-HF-Spanish symptom assessment instrument.

**Methods:**

Following standard forward and backward translation of the original and previously validated English version of the MDASI-HF, a cognitive debriefing with nine native Spanish speaking participants was conducted to evaluate the participants’ understanding and comprehension of the MDASI-HF-Spanish. To examine the comprehensibility, acceptability and psychometric properties of the translated instrument, the MDASI-HF-Spanish was tested in a convenience sample of 50 Spanish speaking patients with a diagnosis of cancer and HF. Evidence for the psychometric validity of the MDASI-HF-Spanish was demonstrated via its internal consistency reliability and known-group validity.

**Results:**

Overall, the participants had no problems with the understandability, readability, or number of questions asked. The MDASI-HF-Spanish subscales showed good internal consistency reliability, with a Cronbach’s coefficient alpha of 0.94 (13 core cancer symptoms), 0.92 (8 heart failure symptoms), and 0.90 (6 interference items) respectively. The MDASI-HF-Spanish was able to differentiate the functional status between patients based on the New York Heart Association (NYHA) functional classification.

**Conclusions:**

The MDASI-HF-Spanish is linguistically and psychometrically valid with ease of completion, relevance, and comprehensibility among the participants, and it can be a useful tool for clinical management and research purposes.

## Introduction

Despite the commitment of the National Institute of Health to include minorities in clinical trials [1] the lack of validated instruments in foreign languages can impede the recruitment and participation of non-English speaking minorities. Particularly, translations are urgently needed in Spanish, the second most common language spoken by the largest and most rapidly growing racial/ethnic minority group in the United States [2]. As of July 1, 2017, the US Census Bureau estimated that there were 58.9 million Hispanics, representing 18.1% of the total population living in the United States, and projected to reach 111.2 million by 2060 (www. Census.gov/popest. Accessed April 2, 2019). An estimated 41 million US residents or 13.4% of the population speak Spanish at home. The majority of Hispanics in the U.S. are of Mexican origin (64%); Puerto Ricans (9%), Cubans (3.4%), and Dominicans (2.8%) constitute other major national groups.

Epidemiologic studies show that Hispanics have excessive rates of diabetes, obesity, dyslipidemia, and metabolic syndrome, in addition to a high incidence of hypertension and ischemic heart disease [3], which make this ethnic group a particularly vulnerable population for the development of heart failure (HF). With the aging and growth of this population group and the high prevalence of risk factors for heart disease and cancer, it is expected that many Hispanics over 65 years of age with cancer may have a concurrent diagnosis of HF.

Hispanics are vulnerable to health care inequalities. The majority of Hispanics face barriers to health care including lack of health insurance, cultural, and language differences. About 8 million Hispanics in the U.S. do not speak English fluently [4]. Language barrier hinders adequate management of patients, and is an important limiting factor for participation in research [5, 6]. To bridge the language barrier and improve symptom management in Spanish-speaking patients with cancer, a Spanish version of the M.D. Anderson Symptom Inventory (MDASI-Sp) was developed at our institution [7]. The MDASI is a validated patient-reported multi-symptom assessment tool that quantifies common cancers and therapy-related symptoms. It contains 13 “core items” representing important symptoms common across all cancer types and 6 items on how these symptoms interfere with major activities of daily functioning [8]. Numerous non-English translated versions of the MDASI have been previously psychometrically validated for its content, construct, reliability, and validity [9–13]. One of the disease-specific modules is the M.D. Anderson Symptom Inventory-Heart Failure (MDASI-HF), specific for patients with cancer and concurrent HF [14]. The MDASI-HF includes the initial 13 core or general cancer items and 6 interference items, with an additional 8 “heart failure specific items” that were determined to be most important by patients with HF. The MDASI-HF has been psychometrically validated in Chinese [15] .To expand the use of the MDASI-HF to Spanish speaking patients with cancer and HF, and to ensure the inclusion of these patients in symptom prevention and intervention clinical studies that use the MDASI-HF as an end-point measure, we translated the MDASI-HF to Spanish. This article describes the linguistic and psychometric validation of the translated Spanish version of the MDASI-HF-Spanish.

## Methodology

Prior to data collection, approval of the study was obtained from the institutional review board (IRB) of the University of Texas MD Anderson Cancer Center. Following IRB approval, adult cancer patients who are > 18 years of age, with a concurrent diagnosis of HF, and able to read and understand Spanish, were recruited from the inpatient and outpatient clinics. Data collection tools included an investigator completed checklist which includes the demographics data form (i.e. age, race, sex, education level, cancer diagnosis, and New York Heart Association (NYHA) Functional classification [16, 17]. The NYHA functional classification places patients in one of four categories - Class I – IV, based on participant’s symptoms during physical activity. The classification is based on participants symptomatology evidence consistent with class I (no limitation of physical activity), class II (slight limitation with ordinary physical activity resulting in fatigue, palpitation, and dyspnea; class III (marked limitation to less than ordinary activity causing fatigue, palpitation or dyspnea); and class IV (unable to carry on any physical activity without discomfort and symptoms of heart failure at rest). Patients with a known diagnosis of dementia or Alzheimer’s disease, who do not speak or write Spanish, and who are unable or refuse to participate were excluded from the study.

A sample of 50 native Spanish speaking cancer patients with a confirmed diagnosis of HF were included in the study, 9 patients for cognitive debriefing, and 41 patients for the psychometric validation of the 8 HF specific items of the MDASI-HF-Spanish. For purposes of this study, native Spanish speaking patients included those who were born in a country that has Spanish as a primary language or live in a home where Spanish is a primary language (as described by the patient). Patients with HF was defined as a having a documented diagnosis of HF in the electronic health record, with left ventricular ejection fraction (LVEF) of < 50% (systolic dysfunction), or an LVEF of > 50% with clinical signs and symptoms of HF (diastolic dysfunction).

### Translation method

The MDASI instrument (13 core symptom items and the 6 symptom interference items) have been previously translated and psychometrically validated in Spanish resulting in a Spanish version (MDASI – Sp) [7] of the instrument. To validate a Spanish version the MDASI-HF, the 8 additional symptoms items specific to HF were translated to Spanish following the linguistic standards of competence through a process of forward and backward translation methodology based on linguistic adaptation principles detailed in the International Society for Pharmacoeconomics and Outcomes Research (ISPOR) [18, 19]. The Spanish bilingual translators were from different Spanish speaking countries of origin including Venezuela, Mexico, Peru, Puerto Rico, and Spain. The use of Spanish colloquial words in English translation may vary with different countries. Because it is impossible to attain 100% equivalence, we strive to minimize bias and come as close to a level of equivalence as possible.

The forward translation was performed by two translators (from Mexico and Venezuela) who spoke Spanish as their native tongue. The forward translation focused on capturing the meaning of the item rather than simply a literal, word-for-word translation. Translators were instructed to use simple language to capture the meaning of the question rather than perform literal translations that are comprehensible for patients with lower reading level. The two forward translations were then submitted to a third independent translator (from Mexico) who resolved existing discrepancies. The reconciled language version then underwent back-translation to the original English version by a fourth translator (from Puerto Rico, with English as the native tongue) not previously involved in the project.

After the initial translation process was completed, six bilingual health professionals (Mexico [2], Venezuela, Puerto Rico, Peru, Spain) and 1 bilingual non-health related professional reviewed the forward, reconciled, and back translations. The reviewers were instructed to work independently and to consider the language that would be understood by patients with diverse educational levels. Reviewers selected the most appropriate translation for each item from the reconciled and independent forward translations, and were asked to provide alternative translations. The recommendations were discussed in detail with the individual reviewers until an agreement was achieved for each of the 8 HF specific symptom items included in the Spanish version of the MDASI-HF. The final version of the 8 HF specific symptom items was then submitted to the bilingual reviewers in the Department of Language Assistance Program of MD Anderson Cancer Center for grammar and spelling verification.

To verify comprehensibility and acceptability of the instrument, the revised version was pretested with 9 Spanish speaking patients from different countries of origin, with cancer and HF diagnosis. Pretesting was used to identify and correct possible translation errors that threaten the equivalence of the final translation [18]. The 8 HF specific items of the MDASI-HF-Spanish were well understood and accepted by the patients. Adaptation of the rigorous double-back translation technique, and pretesting of the translated instrument with Spanish-speaking patients resulted in a Spanish version of the (MDASI-HF-Spanish) instrument.

### Cognitive debriefing

Following forward and backward translation, a cognitive debriefing of the MDASI-HF-Spanish was conducted to test the translated version. Cognitive debriefing is a process by which an instrument or patient questionnaire is actively tested among representatives of the target population and target language group to determine if the respondents understand the questionnaire the same as the original would be understood [20]. Cognitive debriefing interactions with participants were conducted by two research team members who are native Spanish speakers, and who were trained to conduct one-on-one cognitive debriefing interviews [21].

Nine native Spanish-speaking patients with cancer and concurrent HF who met eligibility criteria were asked to review the translated MDASI-HF-Spanish questionnaire. In recruiting for the cognitive debriefing cohort, in addition to meeting the inclusion/exclusion criteria, participants were also chosen based on their country of origin to be representative of more than one country with Spanish as the primary language.

During the cognitive debriefing, the Spanish speaking research team member asked each participant to complete the MDASI-HF-Spanish questionnaire by himself or herself. Moreover, the participants were instructed to read the instrument and circle the words, phrases, or sentences that were difficult to understand. After the participants had completed the questionnaire, the research team member reviewed the form to check for missing data or other problems. Thereafter, during the debriefing interviews, participants were personally interviewed about the instructions, the response format itself and all the instrument items. Participants were asked to explain why any circled words (if any) were difficult to understand, and to paraphrase the sentence in the questionnaire that were difficult to answer. If there are alternative word choices, participants were asked which option they consider more natural. The interviewer judged whether items were correctly paraphrased and recorded any comprehension problems or proposed changes to the wording. The cognitive debriefing process allows for an in-depth interview that may take 30–60 min or more and during which the participant is asked to address each item on the measure. The initial translation to Spanish used “su peor” at the beginning of the sentence. However, one patient from Argentina made a comment that “su peor” has a negative connotation implying “your worst” symptom. After discussion among the members of the translation team, and the Spanish linguistic expert’s consultation, a general consensus was reached to use “Lo peor”. The 8 HF specific symptom items (Table 1) of the MDASI-HF-Spanish were well understood by the participants and were clarified in the cognitive debriefing phase.

**Table 1:** Translation Table Instrument: M.D. Anderson Symptom Inventory – Heart Failure, (MDASI – HF Spanish) Language Pair: English to Spanish

Source Text		Existing Translation	Harmonized Translation	Back-translation	Discussion
1.	Your problem with abdominal bloating at its WORST?	¿ Lo PEOR que se ha sentido cuando tiene problemas de gases abdominales?	NA – No change from existing translation	NA – No change from existing translation	Approved by translators
2.	Your problem with ankle swelling at its WORST?	¿ Lo PEOR que se ha sentido cuando se le inflaman (hinchan) los tobillos?	NA – No change from existing translation	NA – No change from existing translation	Approved by translators
3.	Your difficulty sleeping without adding more pillows under your head at its WORST?	La mayor dificultad cuando ha tenido que dormir sin colocar almohadas adicionales debajo de su cabeza?	NA – No change from existing translation	NA – No change from existing translation	Approved by translators
4.	Your problem with lack of energy at its WORST?	¿ Lo PEOR que se ha sentido cuando le falta energía?	NA – No change from existing translation	NA – No change from existing translation	Approved by translators
5.	Your problem with racing heartbeat (palpitation) at its WORST?	¿ Lo PEOR que se ha sentido cuando tiene palpitaciones?	NA – No change from existing translation	NA – No change from existing translation	Approved by translators
6.	Your problem with nighttime cough at its WORST?	¿ Lo PEOR que se ha sentido cuando tiene tos durante la noche?	NA – No change from existing translation	NA – No change from existing translation	Approved by translators
7.	Your problem with waking up at night with difficulty breathing at its WORST?	20.¿ Lo PEOR que se ha sentido cuando se despierta durante la noche con dificultad al respirar?	NA – No change from existing translation	NA – No change from existing translation	Approved by translators
8.	Your problem with sudden weight gain at its WORST?	¿ Lo PEOR que se ha sentido cuando aumenta de peso repentinamente?	NA – No change from existing translation	NA – No change from existing translation	The initial translation began with “su peor…”. However, one patient made a comment that “su peor…” implied a negative connotation “your worst…” symptom. After discussion and general consensus with research team, it was changed to “Lo peor”.

The MDASI-HF-Spanish symptom items are rated on 0 to 10 numeric scales from 0 meaning “not present” to 10 meaning “as bad as you can imagine”, and the interference items are rated on 0 to 10 numeric scales from 0 meaning “did not interfere” to 10 meaning “interfered completely.” It took 10–15 min for participants to complete the MDASI-HF-Spanish. None of the participants experienced any distress or symptoms during the completion of the instrument.

### Psychometric validation

Although the steps described along with a written report of all decisions made during the development of the MDASI-HF-Spanish are sufficient for linguistic validation per International Society for Pharmacoeconomics and Outcomes Research (ISPOR) [18], additional tests of reliability and validity examined the internal reliability and known-group validity in accordance with accepted linguistic and cross-cultural translation standards [20]. To examine the comprehensibility, acceptability and psychometric properties of the translated instrument, the MDASI-HF-Spanish was tested in a convenience sample of 50 Spanish speaking patients with a diagnosis of cancer and HF. Sample size was based on how precise we want to estimate our Cronbach alpha. Given that there are 8 heart failure specific items on the MDASI-HF, with 50 subjects, if Cronbach’s álpha = 0.9, the 95% confidence interval will extend from 0.85 to 0.94. Internal consistency reliability was assessed using Cronbach’s alpha, and known-group validity was assessed by determining whether MDASI-HF-Spanish scores differ with New York Heart Association (NYHA) functional classification grouping.

## Results

All participants had cancer with a concurrent HF diagnosis, were at least 18 years of age; were able to read and understand Spanish, and provided informed consent. Table 2 identifies demographic characteristics of the participants.

**Table 2:** Demographics of Overall Sample (*n* = 50)

Variable	Frequency	Percent
Sex		
Female	27	54
Male	23	46
Race		
Caucasian	9	18
Other	41	82
Age (years)		
18–50	15	30
51–65	13	26
Over 65	22	44
NYHA Classification		
I	31	62
II	12	24
III	5	10
IV	2	4
Tumor type		
Solid	38	76
Hematologic	12	24

### Cognitive debriefing

The nine patients who underwent cognitive debriefing completed the MDASI-HF-Spanish in approximately 11 min, on average. The nine participants were from varied Spanish-speaking countries of origin including Argentina, Colombia, and Mexico, and different levels of education, from less than high school to a bachelor’s degree. All of the participants reported that the questionnaire was easy to complete, easy to understand, and not repetitive. They were very comfortable answering the questions and had no problems with the understandability, readability, or number of questions asked.

All of the patients found the 0–10 numeric scale easy to use and understand and were comfortable using it. The Flesch-Kinckaid readability score of the English version of the MDASI-HF was at a 4th grade reading level (https://www.webfx.com/tools/read-able/flesch-kincaid.html).

### Psychometric validation

#### Internal consistency reliability

The MDASI-HF-Spanish subscales showed good internal consistency reliability. The data showed Cronbach’s coefficient alpha of 0.94 (13 core cancer symptoms), 0.92 (8 heart failure symptoms), and 0.90 (6 interference items). These values are well above the usual minimum criterion for reliability of 0.70.

#### Known-group validity

Known-group validity comparisons were made for the MDASI-HF-Spanish subscales relative to New York Heart Association (NYHA) functional classification. The MDASI-HF- Spanish differentiated between patients with good versus poor NYHA status: patients with good functional status (NYHA I, II) had lower symptom severity scores for all three subscales than did patients with poor functional status (NYHA III, IV). Because of our small sample size, we did not expect to see a statistically significant result. Instead, we relied on the magnitude of the effect sizes (Table 3). Sloan et al. (2003) have shown that effect sizes of about 0.5 standard deviations can be considered clinically meaningful [21].

**Table 3:** Differences in MDASI-HF-Spanish scales in patients with good vs poor NYHA Functional Classification

NYHA I,II (*N* = 43)			NYHA III,IV (*N* = 7)			
MDASI-HF-Spanish subscale	Mean	Standard deviation	Mean	Standard deviation	Mean Difference	Effect size
Core Symptoms	2.57	2.48	3.88	2.18	1.3	0.56
HF Specific Symptoms	3.10	2.68	4.81	1.97	1.71	0.73
Interference Items	2.22	2.50	3.77	2.02	1.55	0.68

NHYA (New York Heart Association Functional Classification); MDASI-HF-Spanish (Spanish version MD Anderson Symptom Inventory – Heart Failure)

## Discussion

The translation of the MDASI-HF from its original English version to Spanish underwent a rigorous double back translation process combined with cognitive debriefing with representatives from a variety of native Spanish speaking countries. This comprehensive process enabled the development of a high quality Spanish translation of the MDASI-HF instrument that can be used for symptom monitoring, clinical management, and research in Spanish-speaking patients with cancer and HF.

The internal consistency reliability of the MDASI-HF – Spanish is comparable to the Chinese translated version of MDASI-HF- Chinese [15] with the Cronbach’s coefficient alpha for the 13 core items (α = .913), 8 HF items (α = .835), and interference items (α = .897), and with the English validation of the MDASI-HF with a Cronbach’s coefficient alpha of α = .89 (13 core cancer symptoms), α = .83 (8 HF symptoms), and α = .92 (6 interference items) [14], indicating a high internal consistency reliability of the MDASI-HF-Spanish.

Because not all Spanish speaking countries are represented in the linguistic validation of this instrument, it is considered a limitation in this study, especially in the United States, where many versions of Spanish are spoken. Since validation is an iterative process, further testing of the instrument in Spanish speaking countries is encouraged, where the sample sizes are larger and it would be interesting to determine whether the MDASI-HF-Spanish performs as well as in the United States.

## Conclusion

The MDASI-HF-Spanish is linguistically and psychometrically valid, and can be a useful clinical and research instrument for monitoring patients with cancer and HF.

## Data Availability

Not applicable

## Abbreviations

HF: Heart Failure
ISPOR: International Society for Pharmacoeconomics and Outcomes Research
LVEF: Left ventricular ejection fraction
MDASI: MD Anderson Symptom Inventory
MDASI-HF: MD Anderson Symptom Inventory-Heart Failure
MDASI-HF-Spanish: MD Anderson Symptom Inventory - Heart Failure - Spanish version
NYHA: New York Heart Association
